# A Cross-Sectional Study of Tobacco Advertising, Promotion, and Sponsorship in Airports across Europe and the United States

**DOI:** 10.3390/ijerph13100959

**Published:** 2016-09-28

**Authors:** Andrea Soong, Ana Navas-Acien, Yuanjie Pang, Maria Jose Lopez, Esther Garcia-Esquinas, Frances A. Stillman

**Affiliations:** 1 Department of Health, Behavior and Society, Institute for Global Tobacco Control, Johns Hopkins Bloomberg School of Public Health, Baltimore, MD 20742, USA; fstillm1@jhu.edu; 2 Department of Environmental Health Sciences, Johns Hopkins Bloomberg School of Public Health, Baltimore, MD 20742, USA; anavasa1@jhu.edu; 3 Department of Epidemiology, Johns Hopkins Bloomberg School of Public Health, Baltimore, MD 20742, USA; yuanjie.p@gmail.com; 4 Public Health Agency of Barcelona, Barcelona 08023, Spain; mjlopez@aspb.cat; 5 Consortium for Research on Epidemiology and Public Health (CIBERESP), Madrid, Spain; esthergge@gmail.com; 6 Sant Pau Institute of Biomedical Research (IIB Sant Pau), Barcelona 08025, Spain; 7 Department of Preventive Medicine and Public Health, School of Medicine, Universidad Autónoma de Madrid-IdiPaz, Madrid 28220, Spain

**Keywords:** secondhand smoke, tobacco advertising, promotion and sponsorship (TAPS), smoke-free policy, environmental/occupational health

## Abstract

Tobacco advertising, promotion, and sponsorship (TAPS) bans are effective and are increasingly being implemented in a number of venues and countries, yet the state of TAPS in airports and their effect on airport smoking behavior is unknown. The objective of this study was to evaluate the presence of TAPS in airports across Europe and the US, and to begin to examine the relationship between TAPS and smoking behaviors in airports. We used a cross-sectional study design to observe 21 airports in Europe (11) and the US (10). Data collectors observed points of sale for tobacco products, types of products sold, advertisements and promotions, and branding or logos that appeared in the airport. Tobacco products were sold in 95% of all airports, with significantly more sales in Europe than the US. Advertisements appeared mostly in post-security areas; however, airports with advertisements in pre-security areas had significantly more smokers observed outdoors than airports without advertisements in pre-security areas. Tobacco branding appeared in designated smoking rooms as well as on non-tobacco products in duty free shops. TAPS are widespread in airports in Europe and the US and might be associated with outdoor smoking, though further research is needed to better understand any relationship between the two. This study adds to a growing body of research on tobacco control in air transit and related issues. As smoke-free policies advance, they should include comprehensive TAPS bans that extend to airport facilities.

## 1. Introduction

The World Health Organization Framework Convention on Tobacco Control (WHO FCTC) Article 13 and its implementation guidelines call for a comprehensive ban of tobacco advertising, promotion, and sponsorship (TAPS), including in airports and airplanes [1]. Airport smoke-free policies have been implemented indoor in airports in Spain and Russia [2,3], while a California state law requires buildings to restrict outdoor smoking a minimum distance from entrances [4]. However, the widespread availability of tobacco products for sale, the presence of advertisements, and other subtler forms of tobacco promotion and sponsorship detract from these smoke-free policies. In addition to direct advertisements and sales, sponsorship of designated smoking rooms is a well-known tactic that the tobacco industry uses for brand promotion [5,6], and several recent papers have called for their removal [2,7,8]. Research on tobacco promotion and advertising in airports is needed to inform policies that restrict or ban TAPS in airports [9].

The WHO FCTC defines tobacco advertising and promotion as “any form of commercial communication, recommendation or action with the aim, effect or likely effect of promoting a tobacco product or tobacco use either directly or indirectly,” and defines tobacco sponsorship as “any form of contribution to any event, activity or individual with the aim, effect or likely effect of promoting a tobacco product or tobacco use either directly or indirectly” [1]. TAPS are associated with the initiation and continuation of smoking among youth and young adults [10]. For instance, tobacco promotion and advertising in retail environments can influence smoking initiation among adolescents [11,12,13], impulse purchases among smokers [14,15,16], and failed attempts at cessation [17]. The state of TAPS in airports is unknown, and millions of travelers are potentially exposed to pro-tobacco advertisements and messages: Europe was the most visited region overall in 2014 and received 17 million more international tourist arrivals in 2014, while the Americas had an increase of 14 million [18]. Duty free sales, which are ubiquitous in airports, circumvent existing laws that increase taxes on tobacco products [9], and thereby reduce the potential tax revenue that could be used toward tobacco control efforts to reduce cigarette consumption and prevent deaths from tobacco use [9]. Prior research has also highlighted inconsistencies between airport smoking policy and in-country legislation around smoking policy [2].

In this study, we evaluated the presence of TAPS in airports across Europe and the US, and to begin to examine the relationship between TAPS and smoking behaviors in airports using a cross-sectional observational study design. There is a growing body of research around secondhand smoke and smoking policy in airports, and TAPS are an important piece of this topic because of their association with smoking behavior. Understanding more about how much and where TAPS occur in airports could have important implications for building tobacco control efforts.

## 2. Materials and Methods

### 2.1. Study Design and Population

This cross-sectional observational study was conducted by the Institute for Global Tobacco Control and funded by the Flight Attendant Medical Research Institute (FAMRI) to evaluate secondhand smoke exposure, smoking policy, and tobacco product promotion and sales in airports across the US and Europe. Findings on the physical environment of airports and exposure to secondhand tobacco smoke in indoor and outdoor locations are presented in a separate manuscript [19], and findings on e-cigarettes observed in airports and during flights are presented in a separate manuscript [20]. The Institutional Review Board at the Johns Hopkins Bloomberg School of Public Health approved all study protocols and materials, and this study did not involve human subjects.

Data collectors (3 in the US and 5 in Europe, all of them public health professionals) visited a total of 21 large and mid-sized airports (10 US, 11 Europe) between March and May 2014. Eleven airports were in Europe (Amsterdam (AMS), Brussels (BRU), Paris (CDG), Dublin (DUB), Frankfurt (FRA), Rome (FCO), Istanbul (IST), London (LHR), Madrid (MAD), Munich (MUC), Moscow (SVO)), and 10 airports were in the US (Atlanta (ATL), Boston (BOS), Charlotte (CLT), Newark (EWR), Washington DC (IAD), Houston (IAH), New York (JFK), Las Vegas (LAS), Chicago (ORD), Philadelphia (PHL)). Airports were selected using a list of responses provided by flight attendants who were asked in a 2012 survey to list airports where they recalled seeing or smelling cigarette smoke in the past month of traveling [21]. From this list, eight data collectors from the US, Spain, Italy, and Russia were assigned at least two airports to visit on a convenience basis between March and May 2014. Data collectors completed training on observational and study procedures and use of a standardized data collection protocol adapted from the Smoke-Free Compliance Guide [22]. They also participated in a practice round of observations with researcher supervision at a selected airport in Europe (Madrid) and the US (Baltimore).

### 2.2. Data Collection

Airport observations were conducted in one terminal per airport, divided into multiple indoor and outdoor areas (Supplementary Materials Table S1). Within each area data collectors observed multiple locations, defined as stationary points in the airport where travelers and employees may visit or pass through, including but not limited to entrances, walkways, kiosks, restrooms, restaurants, and stores. At each location, data collectors completed a short questionnaire on the TAPS environment in pre- and post-security areas, providing information on what types of products were sold, where they were sold, as well as any advertisements and promotions that appeared. Data collectors were trained to include any type of paraphernalia for sale or otherwise displaying with a tobacco brand name or logo located in airport premises. Each airport was visited once for data collection, and observations lasted approximately two to four hours.

### 2.3. Measures and Variables

Items on the observation form included observation of tobacco products for sale in the terminal (yes/no), where products were sold (duty free shop, kiosk/stand, vending machine, restaurant/café/bar, and other), type of products (smoke tobacco, smokeless tobacco, e-cigarettes), observation of product promotion or advertisements (yes/no), and where the promotion appeared (point of sale, restaurant/café/bar, poster/billboard, other) (Supplementary Materials Table S2). Options for “other” provided an additional space for collectors to fill in qualitative information to describe characteristics of the retail environment, such as store names, and the tobacco brands observed. As part of the parent study on secondhand smoke exposure in airports [19], data collectors counted the number of smokers standing outside of airport entrances or exits. Data were entered from paper forms into an electronic database, and all analyses were performed with Stata (version 13.1, StataCorp, Austin, TX, USA) [23].

### 2.4. Statistical Analysis

We reported the percentage of airports with presence of TAPS in any indoor locations. We calculated the percentage separately for each TAPS item. Data included presence of tobacco for sale by product type (cigarette, cigar/cigarillo, smokeless tobacco), e-cigarettes for sale, and presence of advertisements for tobacco products and e-cigarettes. We reported the findings in pre- and post-security facilities separately and combined in all airports. We then compared presence of TAPS in the same location by region (Europe and US). We reported the mean number of smokers in outdoor locations per airport by presence of tobacco sales and e-cigarette sales in any location in the airport. Outdoor locations included departures and arrivals. Due to the small sample size, we used the Kruskal-Wallis test to compare the distributions, and Fisher’s exact test to compare the percentages. For the Kruskal-Wallis test, we also reported the H statistic and its degree of freedom.

## 3. Results

### 3.1. Tobacco Products

**Figure 1:**
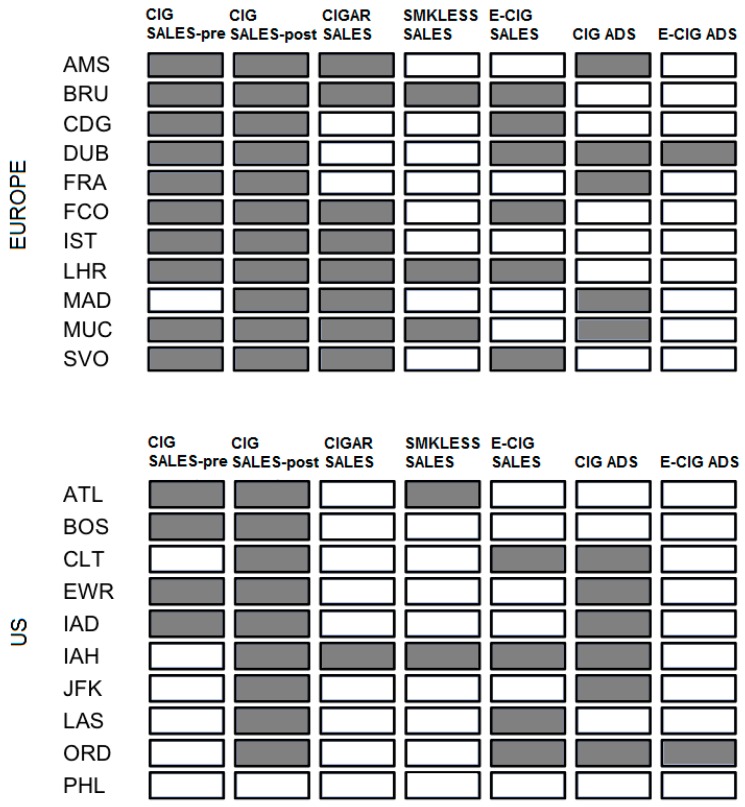
Presence of TAPS in airports in Europe and the US observed in either pre- or post-security locations, 2014 *. 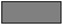, Present; 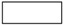, Not present. * Note: Because cigarettes are the most commonly used form of tobacco, the first two data columns depict their presence in pre- and post-security location separately; all other columns depict presence of the product/advertisement in either pre- or post-security location.

Among the 21 airports observed, 14 (67%) sold smoke or smokeless tobacco products in the pre-security area and 20 (95%) in the post-security area (Table 1, Figure 1). Comparing by geographical location, 11 airports (100%) in Europe and 9 (90%) in the US sold smoke or smokeless tobacco products in either pre- or post-security areas. More airports in the US contained post-security cigarette sales and cigarette advertisements (Figure 1). One airport in Europe (DUB) and one in the US (ORD) each had e-cigarette advertisements.

The presence of tobacco products for sale in pre-security areas was significantly lower in the US than in Europe (*p*-value 0.02), and lower in the US but not significantly different from Europe in post-security areas. Tobacco products for sale were most commonly reported to be cigarettes (90%), followed by cigars/cigarillos (43%), and smokeless tobacco (24%). Qualitative responses on types of retail outlets where tobacco products were sold in pre-security areas included various retail spaces in the airports, such as news stands, duty free shops, specialty tobacco shops, and kiosks, as well as a pharmacy and supermarket. For e-cigarettes, six (55%) of the airports in Europe sold them in either pre- or post-security areas. For US airports, four (40%) sold e-cigarettes, all of them in post-security areas.

### 3.2. Tobacco Advertising and Promotion

**Figure 2:**
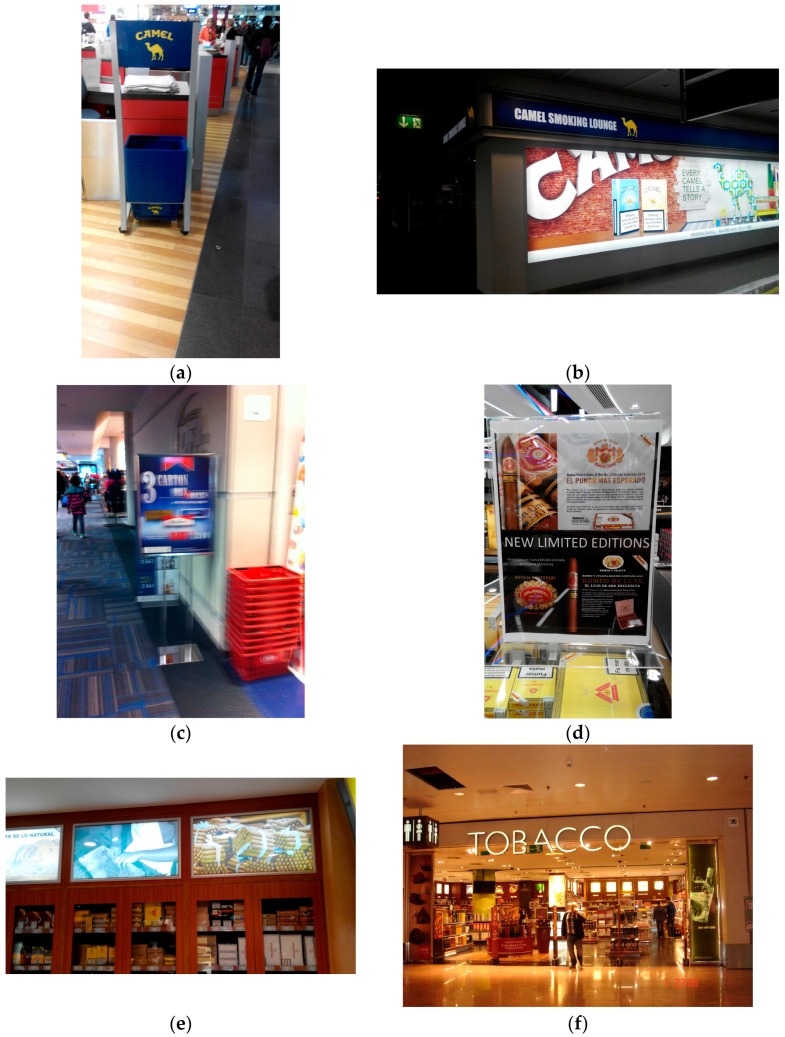
Selected photos from airport observations, 2014. (**a**) Munich airport shopping baskets; (**b**) Munich airport smoking lounge; (**c**) Dulles airport promotion; (**d**) Madrid airport promotion; (**e**) Madrid airport tobacco product display; (**f**) Brussels airport tobacco retail shop.

Tobacco advertisements were observed in 11 airports (52%), with more advertisement in post-security (48%) than pre-security (10%) areas. Tobacco advertisements in Europe were observed in pre-security areas in two airports (18%) and in post-security areas in four airports (36%). In the US, tobacco advertisements were observed in six airports (60%), all of them in post-security areas. E-cigarette advertisements were only present in post-security areas and appeared in two airports. In Munich (MUC), a kiosk used “video ads” promoting Marlboro, and the Camel brand logo appeared on all shopping baskets in the duty free area (Figure 2). Additional photos taken by data collectors of notable advertisements or promotions observed in the airport include a store in Brussels (BRU) specializing exclusively in tobacco products and a placard outside of a store in Dulles (IAD) advertising a discount on cartons of Marlboro.

### 3.3. Evidence of Smoking Behavior

A separate paper discusses in detail the relationship between the physical environment of airports, including presence of smoking behavior or cues indoor and outdoor (smokers, smelling smoke, cigarette butts, ashtrays), presence of designated smoking rooms, no-smoking signage, and other factors [19]. Among the airports containing designated smoking rooms, two rooms in Europe showed cigarette advertisements (Camel brand logo appeared in Frankfurt and Munich, and the Munich room was called the “Camel Smoking Lounge”). One designated smoking room in the US Las Vegas airport showed advertisement of Njoy, an electronic cigarette brand, on the side of a cigarette vending machine.

Analyses revealed a relationship between the presence of tobacco sales and the number of smokers directly outside of airports. Specifically, more smokers were observed outside of airports that sold tobacco products in pre-security areas than in airports that did not sell tobacco products in pre-security areas (data not shown). In seven airports, tobacco products were not observed in the pre-security area, and these airports had a mean of 4.4 (standard deviation 3.8) smokers observed outside. Among the other 14 airports where tobacco products were observed in the pre-security areas, there was a mean of 24.1 (standard deviation 22.6) smokers outside. The difference in the mean number of smokers standing outside airports with and without pre-security tobacco product sales was significant (*p*-value 0.004, H test chi-squared 8.3 (degrees of freedom 1)). In one airport (DUB), the data collector observed a post-security, outdoor smoking area in the form of a restaurant patio, but since this was a unique case these data were not included in the analyses.

## 4. Discussion

Our study found widespread presence of TAPS in major airports across Europe and the US: tobacco products were sold in 95% of all airports, either before or after security checkpoints, with significantly more products for sale overall in Europe than the US. The majority of tobacco products and advertisements appeared in post-security areas, and tobacco paraphernalia such as shopping baskets with tobacco brand logos, colorful advertisements, and other types of artistic displays were ubiquitous. The presence of tobacco industry interference with airport smoke-free policies was apparent in the Camel-sponsored designated smoking rooms in Frankfurt [5,6].

We found a significant relationship between smoking behavior and presence of TAPS at airports, and while these analyses were an exploratory part of a broader parent study of secondhand smoke in airports, the implications are telling. Specifically, airports with tobacco sales in pre-security areas were 3.3 times more likely to have smokers, smoke smell, or cigarette butts indoor, and had a significantly higher number of smokers outdoors; thus, TAPS in pre-security locations may be a facilitator of smoking. One explanation for this could be that travelers who are exposed to TAPS before a flight (pre-security) are perhaps more likely to violate smoke-free policy to satisfy cravings before departure due to stress or anxiety associated with travel. TAPS are associated with the initiation and continuation of smoking among youth and young adults [10]. Due to the large volume of passengers and employees that pass through airports every day, airports may represent a key environment where individuals—particularly youth—can be susceptible to pro-tobacco cues and messages. Given the steadily increasing number of international travelers per year [18], it is essential that policy toward eliminating TAPS in airports be addressed in tobacco control policy.

Although there were more tobacco product advertisements observed in the US than Europe overall, these differences were not statistically significant. Most of the tobacco product sales, advertisements, and promotions that appeared in airports involved smoke or smokeless tobacco products, while e-cigarettes were less common and advertisements appeared only in two airports (Dublin (DUB) and Chicago (ORD)). Interestingly, both of these airports had among the most advanced smoke-free policy in the sample, as neither contained designated smoking rooms, and both restricted smoking at a minimum of 15 feet from the entrances. Data on e-cigarette promotion and retail in airports was novel in this study and additional research could be useful for policymaking as use of the product proliferates globally.

Further research is needed to understand the impact of airport TAPS environments on travelers, such as urge to smoke or seek out a designated smoking room, as this type of relationship is not possible to evaluate with the cross-sectional observational methods used in this study. One approach presented in Article 13 of the WHO FCTC is that retail outlets display only a textual listing of products and prices without advertising any promotional elements [1]. A systematic review of store audit methods for point of sale marketing surveillance provides a useful framework [24], but it is not known whether airports would require a specialized approach due to their unique environment.

Effective implementation of a TAPS ban requires monitoring, enforcement, evaluation, and educational and awareness-raising efforts [25]. Point of sale display bans achieved high compliance and public support in Ireland and Norway [26], and in Thailand, an evaluation of its 2005 ban found high support among smokers [16]. A study of public support of marketing and point of sale bans in the United States found low support [27], and an evaluation of a point of sale ban is currently underway in the United Kingdom [28]. Prior research has found that airports are not necessarily subject to in-country or local smoke-free legislation [2], thus a concerted effort among smoke-free advocates and policy makers may be required to implement a TAPS ban.

The airports surveyed were broadly representative of larger airports in the US and Europe that flight attendants previously reported to have high secondhand smoke exposure, but readers should take caution in applying these findings to all airports of these regions or airports in other geographical areas. For example, the US airports were skewed to the eastern half of the country with only one in the west, Las Vegas, which is arguably the most permissive smoking environment in the Western region. Tobacco use is high in other regions such as Asia and tobacco control policy is less advanced in other regions [29]. Considering these limitations of the study, there is a need to conduct future observational studies in airports in other regions of the world. Comprehensive tobacco promotion and retail display bans are particularly important in lower income countries [30]. It is possible that airports in these countries—as well as smaller, local airports—may have differing policies and enforcement than the airports studied here. Another strength of this research, however, is that the airports visited were all major international hubs, the majority of them among the busiest in the world [31].

Another limitation to consider is potential variation in the day of week or time of day that observations were conducted between airports. Data collectors were required to complete their observations during typical hours in which airport stores and facilities would be open, approximately 8:00 a.m. to 8:00 p.m., but study protocol did not require that observations be completed on a weekday or weekend. It is possible that data could be affected by whether the collection was conducted during a time in which more or less travelers or smokers are typically present.

## 5. Conclusions

The results of this study point toward a possible relationship between TAPS and smoking behavior in airports, and further research is needed to better understand this finding and to inform policies that will encourage the removal of sales and promotion of tobacco products from airports. This study could serve as a model for subsequent studies observing tobacco product retail in similar types of facilities, including transportation hubs. Smoke-free policies should include comprehensive bans on tobacco product promotion and advertising that extend to airport retail outlets. Such policies may prevent travelers, including children and adolescents, from being exposed to pro-tobacco messages, as well as remove cues that prompt smokers or former smokers to purchase or use tobacco. Lastly, more research is needed to help guide implementation and evaluation of effective tobacco control policies, including the elimination of TAPS in airports around the world [25].

## Figures and Tables

**Table 1:** Presence of TAPS observed in airports in Europe and the US, by location, 2014.

TAPS Item	European Airports		US Airports		All Airports	
	Pre-Security	Post-Security	Pre-Security	Post-Security	Pre-Security	Post-Security
	% (*n*)	% (*n*)	% (*n*)	% (*n*)	% (*n*)	% (*n*)
Sales present	91 (10)	100 (11)	40 * (4)	90 (9)	67 (14)	95 (20)
Cigarette	82 (9)	55 (6)	40 (4)	70 (7)	62 (13)	62 (13)
Cigar/cigarillo	64 (7)	36 (4)	0 * (0)	10 (1)	33 (7)	24 (5)
Smokeless	18 (2)	9 (1)	10 (1)	10 (1)	14 (3)	10 (2)
E-cigarettes	36 (4)	45 (5)	0 (0)	36 (4)	19 (4)	38 (9)
Advertisements present	18 (2)	36 (4)	0 (0)	60 (6)	10 (2)	48 (10)
Tobacco products	18 (2)	36 (4)	0 (0)	60 (6)	10 (2)	48 (10)
E-cigarettes	0 (0)	9 (1)	0 (0)	10 (1)	0 (0)	10 (2)

* *p* < 0.05.

## Supplementary Materials

The supplementary materials are available online at www.mdpi.com/1660-4601/13/10/959/s1. Table S1. Overview of observation locations and procedures; Table S2. Sample of tobacco product promotion and sales data collection procedure.
