# Supplementary material for: A Cross-Sectional Study of Tobacco Advertising, Promotion, and Sponsorship in Airports across Europe and the United States
Source: Int J Environ Res Public Health. 2016 Sep 28;13(10):959. doi: 10.3390/ijerph13100959 (PMC5086698; doi:10.3390/ijerph13100959)
Supplement: Supplementary file 1 [file ijerph-13-00959-s001.pdf]

Supplementary Materials: A Cross-Sectional Study of Tobacco Advertising, Promotion, and Sponsorship in Airports across Europe and the United States

Andrea Soong, Ana Navas-Acien, Yuanjie Pang, Maria Jose Lopez, Esther Garcia-Esquinas and Frances A. Stillman

Table S1. Overview of observation locations and procedures.

Section of Airport	Maximum Number of Locations to Observe	Additional Data Collection Questions
Departures, outdoor	6	3
Departures, indoor	6	2
Shops and facilities, pre-security	10	2
Tobacco product promotion and sales, pre-security	1	6
Shops and facilities, post-security	20	2
Tobacco product promotion and sales, post-security	1	6
Designated smoking room (DSR)	1	17
Business lounge	1	8
In-flight observations	1	9
Arrivals, indoor	6	2
Arrivals, outdoor	6	3

Table S2. Sample of tobacco product promotion and sales data collection procedure.

**Name of Airport:** ________________________	**Observer ID:** ___ ___/___ ___
**3-Letter Code:** ________________________	*Initials* *ID #*
**Terminal:** ________________________	**Date:** __ __/__ __/__ __ __ __<br>*DD MM YYYY*

1. Do you observe any tobacco products for sale in the terminal? Ask an airport employee if necessary. ❑ Y ❑ N

A If yes, specify where the products are sold (check all that apply).

❑ Duty free shop ❑ Kiosk/stand ❑ Vending machine ❑ Restaurant/café/bar ❑ Other, specify: _________

B. Specify the type of products for sale (check all that apply).

❑ Cigarettes ❑ Cigars/cigarillos ❑ Smokeless tobacco ❑ Other, specify: _________

2. Do you observe any tobacco product promotions or advertisements in the terminal? ❑ Y ❑ N

A. Describe where the promotion appeared (check all that apply).

❑ Point of sale ❑ Restaurant/café/bar ❑ Poster/billboard ❑ Other, specify: _________

3. If possible, ask an airport employee where you can smoke in the airport. What did he/she say?

____

____

4. Do you observe any e- cigarettes for sale in the terminal? Ask an airport employee if necessary. ❑ Y ❑ N

A. If yes, specify where they are sold (check all that apply).

❑ Duty free shop ❑ Kiosk/stand ❑ Other, specify: ________________________

B. Name the *first* brand of e-cigarette that you see for sale: ____

C. For this brand, what is the price of: ❑ Starter kit: _______ ❑ Refill cartridge: _______ (**Indicate currency**: _______)

5. Do you observe any e-cigarette product promotions or advertisements in the terminal? ❑ Y ❑ N

A. Describe where the promotion appeared.

❑ Point of sale ❑ Restaurant/café/bar ❑ Poster/billboard ❑ Other, specify: ________

6. If possible, ask an airport employee where you can use e-cigarettes in the airport. What did he/she say?

© 2016 by the authors. Submitted for possible open access publication under the terms and conditions of the Creative Commons Attribution (CC-BY) license (http://creativecommons.org/licenses/by/4.0/).
